# Effectiveness of workshops to teach a home-based exercise program (BEST at Home) for preventing falls in community-dwelling people aged 65 years and over: a pragmatic randomised controlled trial

**DOI:** 10.1186/s12877-022-03050-2

**Published:** 2022-04-26

**Authors:** Amanda Bates, Susan Furber, Cathie Sherrington, Paul van den Dolder, Karen Ginn, Adrian Bauman, Kirsten Howard, Michelle Kershaw, Lisa Franco, Cathy Chittenden, Anne Tiedemann

**Affiliations:** 1 Health Promotion Service, Illawarra Shoalhaven Local Health District, Wollongong, NSW Australia; 2 Illawarra Health and Medical Research Institute, Wollongong, NSW Australia; 3 School of Health and Society, Faculty of the Arts, Social Sciences and Humanities, University of Wollongong, Wollongong, NSW Australia; 4 Institute for Musculoskeletal Health, The University of Sydney and Sydney Local Health District, Sydney, NSW Australia; 5 School of Public Health, Faculty of Medicine and Health, The University of Sydney, Sydney, NSW Australia; 6 Primary Care and Community Health, Nepean Blue Mountains Local Health District, Kingswood, NSW Australia; 7 School of Medical Sciences, Faculty of Medicine and Health, The University of Sydney, Sydney, NSW Australia; 8 Department of Physiotherapy, Illawarra Shoalhaven Local Health District, Wollongong, NSW Australia

**Keywords:** Accidental falls, Aged, Exercise, Falls prevention, Randomised controlled trial

## Abstract

**Background:**

Falls are a significant public health issue. There is strong evidence that exercise can prevent falls and the most effective programs are those that primarily involve balance and functional exercises, however uptake of such programs is low. Exercise prescribed during home visits by health professionals can prevent falls however this strategy would be costly to deliver at scale. We developed a new approach to teach home exercise through group-based workshops delivered by physiotherapists. The primary aim was to determine the effect of this approach on the rate of falls among older community-dwelling people over 12 months. Secondary outcomes included the proportion of people falling, fear of falling, physical activity, lower limb strength, balance and quality of life.

**Methods:**

A randomised controlled trial was conducted among community-dwelling people aged ≥65 in New South Wales, Australia. Participants were randomised to either the intervention group (exercise targeting balance and lower limb strength) or control group (exercise targeting upper limb strength).

**Results:**

A total of 617 participants (mean age 73 years, +SD 6, 64% female) were randomly assigned to the intervention group (*n* = 307) or control group (*n* = 310). There was no significant between-group difference in the rate of falls (IRR 0.91, 95% CI 0.64 to 1.29, *n* = 579, *p* = 0.604) or the number of participants reporting one or more falls (IRR 0.99, 95% CI 0.76 to 1.29, *n* = 579, *p* = 0.946) during 12 month follow-up. A significant improvement in the intervention group compared to control group was found for fear of falling at 3, 6 and 12 months (mean difference 0.50, 95% CI 0.2 to 0.8, *p* = 0.004; 0.39, 95% CI 0.001 to 0.8, *p* = 0.049; 0.46, 95% CI 0.006 to 0.9, *p* = 0.047, respectively), and gait speed at 3 months (mean difference 0.09 s, 95% CI 0.003 to 0.19, *p* = 0.043). No statistically significant between-group differences were detected for the other secondary outcomes.

**Conclusions:**

There was no significant intervention impact on the rate of falls, but the program significantly reduced fear of falling and improved gait speed. Other exercise delivery approaches are needed to ensure an adequate intensity of balance and strength challenge and dose of exercise to prevent falls.

## Introduction

Falls are a significant and increasing public health issue. In New South Wales (NSW), Australia, more than 25% of people over the age of 65 years fall at least once each year [1]. Falls are one of the most common causes of injuries among older people [2]. Consequences of falls include serious injury, hospitalisation [1], fear of falling [3] and reduced quality of life [4].

A recent Cochrane systematic review and meta-analysis [5] found strong evidence that exercise programs can reduce the rate of falls and that the most effective exercise programs are those that primarily involve balance and functional exercises. The World Health Organisation (WHO) Guidelines on Physical Activity and Sedentary Behaviour (2020) recommend that older adults do functional balance and strength training on three or more days per week, to enhance functional capacity and prevent falls [6]. Many older adults do not meet the exercise recommendations to prevent falls [7]. There is a need to promote uptake and ongoing participation in exercise programs with balance and strength components that are easily accessible to older adults.

Exercise programs for older people can be effective in preventing falls if delivered in either a group or individual format [8, 9]. Individual programs that are carried out at home have been reported to be more appealing to some older people due to their greater convenience, accessibility and lower cost [10, 11]. People who are older and at a higher risk of falls are more likely to prefer home-based exercise programs compared to group-based classes [11, 12]. Home-based strength and balance training has also been shown to be safe and effective in improving balance and strength [13–15]. The Otago Exercise Programme is an effective home-based fall prevention program involving strength and balance exercises [13], which reduces the number of falls and injuries from falls in older community-dwelling adults [13, 16, 17]. The Otago Exercise Programme was originally delivered by physiotherapists or trained community nurses via individual home visits. Bates and colleagues piloted a new method of delivery for the Otago Exercise Programme that used group sessions to deliver the exercise instruction instead of individual home visits [18]. This pilot program increased strength and balance and non-significantly reduced falls in a pre-post study (no control group) [18]. This pragmatic approach that could be scaled up for broader implementation warranted further evaluation in a larger study with a control group.

The primary aim of the current study was to determine the effectiveness of a home-based exercise program (BEST at Home – lower limb) taught through workshops in community venues delivered by physiotherapists and aimed at preventing falls among community-dwelling people aged 65 years and over. The secondary aims were to determine the effect of the BEST at Home program on the proportion of people falling, fear of falling, physical activity, lower limb strength, balance, attitudes to exercise, quality of life, cost-effectiveness and to describe the program acceptability to participants.

## Methods

### Study design

We conducted a pragmatic randomised controlled trial with two parallel arms. After completing the baseline assessment and questionnaires, participants were randomly assigned to either the intervention (lower limb) or control (upper limb) exercise program. Randomisation order was determined using a computer-generated random number schedule (hosted on REDCap) with variable block sizes of 2–6, developed by an investigator not involved in recruitment for the trial. People living in the same household were treated as one unit and allocated to the same exercise program to avoid possible contamination of interventions. Due to the nature of the exercise program, participants and program providers were unable to be blinded to group allocation. Data for the primary outcome of falls was self-reported by participants, however the person following up primary outcome data with participants was blinded to group allocation. Secondary outcomes were collected by assessors who were blinded to the group allocation. Participants were instructed not to inform the assessors of their intervention group. Ethical approval was obtained from the University of Wollongong and Illawarra Shoalhaven Local Health District Human Research Ethics Committee (HE14/279 and HREC/14/WGONG/50). All methods were performed in accordance with the relevant guidelines and regulations. A detailed protocol describing the design and methods of the study has been published [19]. The study reporting is in accordance with the Consolidated Standards of Reporting (CONSORT) [20]. The trial was registered with the Australian and New Zealand Clinical Trials Registry (ACTRN12615000865516) on 19/08/2015.

### Participants

Participants were community-dwelling adults aged 65 years and older residing in the Illawarra and Shoalhaven Local Health District, New South Wales, Australia. They were recruited using a variety of methods, including paid advertisements in local newspapers, media releases, radio interviews, distribution of flyers and other printed material promoting the study and presentations to community groups. Inclusion criterion was community-dwelling and aged 65 years or over. Participants were screened for eligibility over the telephone and were considered ineligible if they had any of the following: cognitive impairment (assessed by a Memory Impairment Screen score of less than 5) [21]; inability to walk 10 m despite assistance from a walking aid; insufficient English language skills to read and understand program materials; a progressive neurological disease (e.g. Parkinson’s disease, multiple sclerosis); fracture or joint replacement within the last 6 months; a medical condition precluding exercise (e.g. unstable cardiac disease, uncontrolled hypertension, uncontrolled metabolic diseases); unable to obtain medical clearance (as determined by their General Practitioner) and currently participating in an exercise program two or more times per week that is similar to either the upper limb or lower limb exercise program.

#### Intervention group

Participants allocated to the intervention group received a home-based exercise program to improve balance and strength in the lower body. This program was based on the Otago Exercise Programme [13, 22] and included 17 balance and strength exercises, such as knee extension and knee flexion, hip abduction, calf raises, toes raises, sit to stand, semi squats from a standing position, tandem stand, tandem walk, sideways walking, backwards walking, heel walking, toe walking, one leg stand, and walking and turning around. Participants were instructed to perform 10–20 repetitions of the exercises, three times per week at home. Participants were provided with an ankle cuff weight (0.5 kg – 5 kg), with the weight determined by the physiotherapist at the first session. A home exercise manual containing diagrams and descriptions of the exercises and a copy of ‘Staying Active and On Your Feet’, a booklet produced by NSW Health about preventing falls [23] was provided to all participants. Participants were shown how to make each exercise progressively more difficult and were encouraged to make the balance exercises more challenging as they continued the program (see Table 1 TIDieR checklist).

**Table 1:** Intervention description using the Template for Intervention Description and Replication (TIDieR) checklist

Checklist item	
1. Brief name	Balance Exercise Strength Training (BEST) at Home (lower limb) trial
2. Why	Falls are a major and increasing public health issue. More than 25% of people 65 years and over fall at least once each year. Balance and strength training has been shown to reduce the risk of falling in older people.
3. What materials	Participants in the intervention group received: - an exercise program designed to improve balance and strength in the lower limbs (including exercise instruction, printed manual and weights); - a booklet on preventing falls titled ‘Staying active and on your feet’ Participants in the control group received: - an exercise program designed to improve upper limb strength and mobility (including exercise instruction, printed manual, weights and exercise band)
4. What procedures	Both the intervention and control groups received three group-based exercise instruction sessions and three measurement sessions.
5. Who provided	Physiotherapists delivered the exercise instruction. Physiotherapists and exercise physiologists conducted the measurements.
6. How	The exercise instruction was delivered face to face in small groups of approximately 10 participants.
7. Where	In the community of the Illawarra and Shoalhaven regions, NSW, Australia.
8. When and how much	Exercise instruction sessions were held in weeks 1, 4 and 12 (1 h duration). Participants were asked to perform the exercises three times per week for 12 months. The first measurement session occurred at baseline before the participant was randomised. The second and third measurement sessions were held at 12 weeks and 6 months. Final questionnaires were posted to participants at 12 months.
9. Tailoring	Exercises were tailored by the physiotherapist for each participant, to meet their level of ability.
10. Modifications	No modifications were made.
11. How well (planned)	Adherence to the exercise program was assessed by self-reported exercise sessions, which were marked on calendars (and returned monthly)
12. How well (actual)	Participants were asked to perform the exercises 3 times per week. Participants in the intervention group completed an average of 94 sessions over the 12 month period (less than twice per week). Participants in the control group completed an average of 104 sessions over the 12 month period (twice per week).

#### Control group

The control group received an upper limb exercise program (BEST at Home – upper limb) designed by members of the research team to improve upper limb strength, mobility, and function. The upper limb exercise program was a set of eight exercises, which included arm raises, internal and external shoulder rotation, elbow flexion and extension, shoulder press, chest press and shoulder row. A pair of dumbbell weights (600 g – 3 kg) and an elastic exercise band (light, medium, heavy or extra heavy resistance) were provided, with the exercise level determined by the supervising physiotherapist. A home exercise program manual containing diagrams and descriptions of the exercises was provided. Participants were instructed to perform 10 repetitions of each exercise during three exercise sessions per week at home. All exercises in the upper limb program were performed in a seated position, to reduce the likely impact on balance and fall prevention and hence to reduce the contamination effect with respect to the lower limb exercise program.

#### Both groups

Both exercise programs were delivered by two experienced physiotherapists in three group workshops. The workshops were run in local community centres or clubs and contained approximately 10 participants. Exercise instruction workshops occurred at weeks 1, 4 and 12, and were one hour in duration. The program was tailored to each participant’s level of ability. At each workshop, the exercises were reviewed, techniques were corrected, and exercises adjusted or progressed by the supervising physiotherapist, according to the ability of each participant.

### Outcome measures

The primary outcome was the rate of falls, recorded with monthly calendars for a 12 month period, post-randomisation [24]. A fall was defined as ‘an unexpected event in which the participants come to rest on the ground, floor, or lower level’ [24]. Falls were recorded using monthly calendars for a 12-month period after randomisation. Calendars were returned in reply paid, preaddressed envelopes. Participants who did not return their calendars were telephoned to ask about falls for that month. Participants who reported a fall were telephoned to confirm the fall and obtain details about fall location, resulting injuries and what treatment was sought. There were several secondary outcomes. Fear of falling was assessed using the short form Falls Efficacy Scale International (FES-I) [25, 26]. Quality of life was assessed with the self-report SF12v2 [27]. Lower limb strength and balance were assessed with the Short Physical Performance Battery (SPPB) [28], the alternate step test [29] and a knee extension (quadriceps) strength test [30]. The assessments of strength and balance were conducted by Physiotherapists and trained research assistants who were blinded to group allocation. Physical activity (including daily step count, counts per minute and minutes of moderate to vigorous physical activity) was measured with an Actigraph accelerometer (model wGT3x-BT) worn at the waist [31, 32]. Accelerometer data were collected over a one-week period to account for day-to-day variation in physical activity levels. Acceptable wear time was defined as a minimum of 4 days of 10 h or more per day. Activity counts per second were collected at a sampling frequency of 30 Hz and reintegrated to 60s epochs for data analysis. Physical activity was also measured using self-report data from the Incidental and Planned Exercise Questionnaire [33]. Paper-based questionnaires were self-completed during sessions at baseline, 12 weeks and 6 months; and via paper postal questionnaires at 12 months. The proportion of people falling in the intervention and control groups was determined by the monthly falls calendars.

Participants also completed a baseline questionnaire that included questions about sociodemographic details, number of prescription medications, number of comorbidities, history of falls, fear of falling (short FES-I), and self-rated balance perception. Attitudes to exercise were assessed by selected questions from the Physical Activity Stages of Change questionnaire, the Exercise Self-Efficacy Scale and the Physical Activity Enjoyment Scale [34–36]. In order to measure program adherence, participants were asked to record the days that they completed the exercises on the falls calendars that they returned each month.

### Data analysis

The number of falls per person-year were analysed using negative binomial regression models to estimate the between-group difference in fall rates after 1 year (primary outcome). Days of follow up was included as an exposure variable in the negative binomial regression analysis. Modified Poisson regression models were used to compare groups on dichotomous outcome measures (proportion of fallers). Linear regression models were used to assess the effect of group allocation on the continuously-scored measures of strength, balance, physical activity (self-report and accelerometer), quality of life (SF-12), fear of falling (short FES-I), after adjusting for baseline scores. For variables that were not normally distributed (short FES-I, SF-12, moderate vigorous physical activity (MVPA), planned physical activity, total walking, SPPB, standing balance, single leg stance) change scores from baseline to follow up were analysed. Separate linear regression analyses were performed for each time point for continuous measures. Interaction terms in the model were used to assess for differential intervention effects by age, sex (male versus female), upper limb dysfunction or previous falls. Statistical significance was set at *p* < 0.05. The data analysis for the primary and secondary outcomes was undertaken blinded to the group allocation and used an intention-to-treat approach. Sample size calculations suggested that 576 participants would provide 80% power to detect as significant, at the 5% level, a 30% lower rate of falls for the intervention group participants than the control group participants (i.e., incidence rate ratio (IRR) = 0.70) with a 15% loss to follow up. The sample size calculation used the nbpower user written command in Stata. StataCorp. 2017 (Stata Statistical Software: Release 15. College Station, TX: StataCorp LLC). We assumed the upper limb group rate of falls to be 0.85 falls/person year over the 12-month follow-up, which is comparable with the fall rates found in similar trials with community based samples [37]. A sensitivity analysis was conducted for the primary outcome to account for the clustering of household participants.

## Results

### Participants

**Fig. 1:**
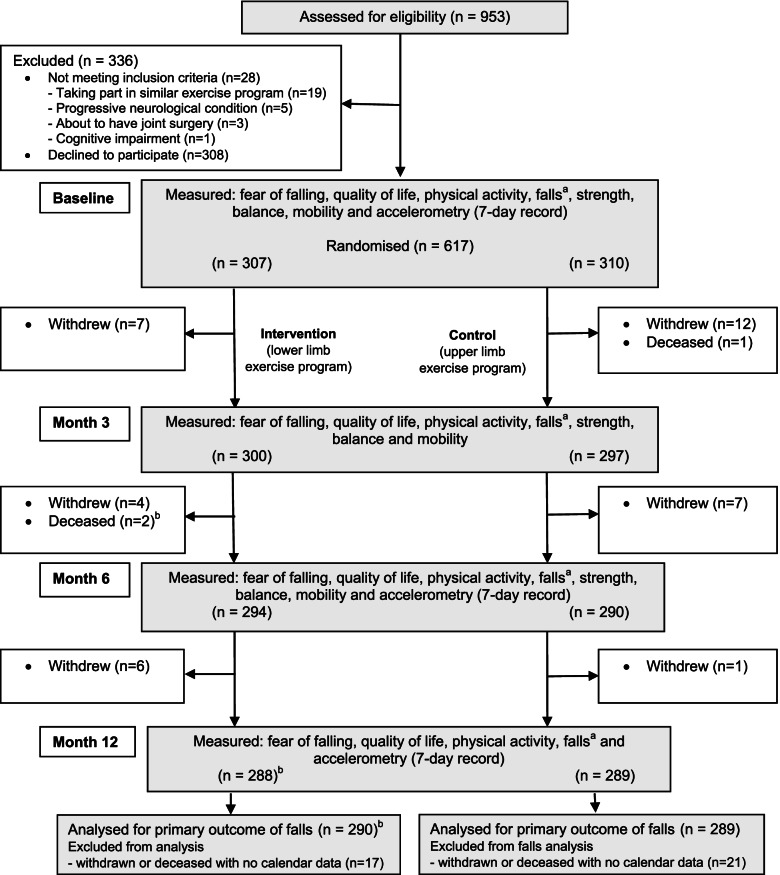
Design and flow of participants through the trial. ^a^Falls were measured monthly. ^b^The two people who died in the intervention group had completed monthly calendars and their data was included in the analysis (*n* = 290)

Recruitment occurred between September 2015 and May 2017. Follow up questionnaires were completed in May 2018. The flow of participants through the study is shown in Fig. 1. A total of 953 participants were screened for eligibility, 308 declined to participate and 28 did not meet the inclusion criteria. A total of 617 participants (mean age 73.1 years, SD 6.0, 63.7% female) were randomly assigned to the intervention group (*n* = 307) or control group (*n* = 310). Baseline characteristics of participants is presented in Table 2. The participants in the two groups were well-matched at baseline (Table 2).

**Table 2:** Characteristics of participants at baseline

Characteristics	Intervention	Control	All
Age (years), mean (SD)	72.9 (6.2) *n* = 307	73.2 (5.8) *n* = 310	73.1 (6.0) *n* = 617
Female: *n (%)*	196 (63.8) *n* = 307	197 (63.6) *n* = 310	393 (63.7) *n* = 617
Lives alone: *n (%)*	89 (29.0) *n* = 307	97 (31.3) *n* = 310	186 (30.2) *n* = 617
Fallen in the past 12 months: *n (%)*	80 (26.1) *n* = 307	86 (27.7) *n* = 310	166 (26.9) *n* = 617
Self-rated balance fair/poor: *n (%)*	99 (32.6) *n* = 304	96 (31.2) *n* = 308	195 (31.9) *n* = 612
Self-rated fear of falling ≥ moderate: *n (%)*	79 (25.7) *n* = 307	72 (23.4) *n* = 308	151 (24.6) *n* = 615
Total medications (n), mean (SD)	3.0 (2.6) *n* = 304	3.3 (2.7) *n* = 305	3.1 (2.7) *n* = 609
Medical conditions (0–17)^a^, mean (SD)	2.8 (1.9) *n* = 307	2.8 (2.0) *n* = 310	2.8 (1.9) *n* = 617
Arthritis: *n (%)*	173 (58.3) *n* = 297	181 (58.6) *n* = 309	354 (58.4) *n* = 606
Osteoporosis: *n (%)*	66 (21.7) *n* = 304	57 (18.6) *n* = 307	123 (20.1) *n* = 611
Diabetes: *n (%)*	26 (8.5) *n* = 306	32 (10.4) *n* = 307	58 (9.5) *n* = 613
Depression: *n (%)*	51 (16.8) *n* = 303	48 (15.8) *n* = 303	99 (16.3) *n* = 606
Self-report physical activity, hours/week^b^: mean (SD)	33.4 (19.1) *n* = 306	34.3 (19.2) *n* = 309	33.9 (19.1) *n* = 615
SF12v2: physical composite score	48.0 (7.7) *n* = 304	47.8 (7.4) *n* = 298	47.9 (7.6) *n* = 602
SF12v2: mental composite score	53.4 (5.7) *n* = 304	53.6 (5.4) *n* = 298	53.5 (5.6) *n* = 602
Short FES-I, mean (SD)	9.7 (3.3) *n* = 307	9.4 (2.9) *n* = 309	9.6 (3.1) *n* = 616
Average daily step count, steps, mean (SD)	5725.9 (2424.6) *n* = 297	5539.6 (2394.5) *n* = 288	5634.2 (2409.5) *n* = 585
MVPA, minutes/day, mean (SD)	18.9 (17.4) *n* = 297	17.2 (16.8) *n* = 288	18.0 (17.1) *n* = 585

*Abbreviations*: *FES-I* Falls Efficacy Scale-International, *MVPA* moderate vigorous physical activity

^a^Possible medical conditions included: arthritis, osteoporosis, asthma, chronic obstructive pulmonary disease, angina, heart disease, heart attack, neurological disease, stroke/transient ischaemic attack, peripheral vascular disease, diabetes mellitus, upper gastrointestinal disease, depression, anxiety/panic disorder, visual impairment, hearing impairment, degenerative disc disease

^b^Measured using the Incidental and Planned Exercise Questionnaire (IPEQ)

### Primary outcome

During the 12 month study period, 157 people (27% of participants who returned at least one calendar) reported 267 falls. In the intervention group 79 participants reported 138 falls and in the control group 78 participants reported 129 falls. There was no difference between the rate of falls in the intervention group compared to the control group (IRR 0.91, 95% CI 0.64 to 1.29, *p* = 0.604, *n* = 579). A sensitivity analysis conducted to adjust for clustering of households found similar results, with no difference in the rate of falls in the intervention group compared to the control group (IRR 0.91, 95% CI 0.63 to 1.32, *p* = 0.624, *n* = 579). Frequencies and percentages of self-reported falls are presented in Table 3. Participants returned an average of 10 months of calendars. A total of 424 (69%) participants completed all 12 months of falls calendars.

**Table 3:** Number of participants falling and total number of falls during 12 month follow up

Falls	All (***n*** = 579)	Intervention (***n*** = 290)	Control (***n*** = 289)	Unadjusted IRR* (95% CI)
Number of falls, n	267	138	129	0.91 (0.64 to 1.29), *p* = 0.604^**a**^
Falls per participant, mean (SD), median (min-max)	0.46 (1.03), 0 (0–12)	0.48 (1.14), 0 (0–12)	0.45 (0.91), 0 (0–5)	
Frequency of falls, n (%)				
0	422 (72.9)	211 (72.8)	211 (73)	
1	102 (17.6)	51 (17.6)	51 (17.6)	
2	27 (4.7)	16 (5.5)	11 (3.8)	
3	14 (2.4)	5 (1.7)	9 (3.1)	
≥ 4	14 (2.4)	7 (2.4)	7 (2.4)	
1+ falls	157 (27.1)	79 (27.2)	78 (27)	0.99 (0.76 to 1.29), *p* = 0.946^b^
Follow up, days, mean (SD)	311.8 (103.6)	306.6 (105.7)	317.1 (101.4)	
Falls indoors, n (%)	90 (33.7)	50 (36.2)	40 (31)	
Falls outdoors, n (%)	177 (66.3)	88 (63.8)	89 (69)	
Falls with fractures, n (%)	16 (6)	12 (8.7)	4 (3.1)	
Falls requiring hospital admission, n (%)	10 (3.7)	5 (3.6)	5 (3.9)	

^a^Between-group difference from negative binomial regression models comparing rates between groups adjusted for exposure: days of follow-up

^b^Between-group difference from Poisson regression models comparing proportions between groups

^*^IRR Incidence rate ratio

^**^38 participants with no calendar data removed for analysis

### Secondary outcomes

Table 4 shows the baseline and follow-up scores for the secondary outcomes. A significant improvement in the intervention group compared with the control group was found for fear of falling (measured by the FES-I) at 3, 6 and 12 months (mean difference = 0.50, 95% CI 0.2 to 0.8, *p* = 0.004; mean difference = 0.39, 95% CI 0.001 to 0.8, *p* = 0.049; mean difference = 0.46, 95% CI 0.006 to 0.9, *p* = 0.047), and gait speed (measured by the 4 m walk) at 3 months (mean difference = 0.09 s, 95% CI 0.003 to 0.19, *p* = 0.043). There were no significant between-group differences in physical activity (accelerometer and self-report), quality of life (SF-12), SPPB, sit to stand, balance, alternate step test, leg strength and gait speed (at 6 months). There was no difference in the proportion of people falling in the intervention and control groups (IRR 0.99, 95% CI 0.76 to 1.29, *p* = 0.946), as demonstrated by the number of participants reporting one or more falls during the 12 month follow up (Table 3). The cost-effectiveness of the intervention will be reported separately.

**Table 4:** Intervention effects on secondary outcomes

Outcome measure	Intervention Mean (SD), ***n***	Control Mean (SD), ***n***	Mean difference (95% CI)	***P*** value
**Falls Efficacy Scale-International (7–28)**^**b c**^				
Baseline	9.7 (3.3) *n* = 307	9.4 (2.9) n = 309		
3 months	9.1 (2.7) *n* = 262	9.2 (2.7) *n* = 250	0.50 (0.2–0.8)	0.004*
6 months	9.2 (2.8) *n* = 243	9.3 (2.9) *n* = 250	0.39 (0.001–0.8)	0.049*
12 months	9.1 (2.7) *n* = 231	9.4 (2.9) *n* = 235	0.46 (0.006–0.9)	0.047*
**Quality of life – physical (SF12 physical component summary score)**^**a c**^				
Baseline	48.0 (7.7) *n* = 304	47.8 (7.4) *n* = 298		
3 months	48.5 (7.6) *n* = 257	48.1 (7.0) *n* = 243	−0.28 (−1.2–0.6)	0.534
6 months	48.0 (8.2) *n* = 238	47.7 (7.8) *n* = 244	0.26 (− 0.8–1.3)	0.633
12 months	48.5 (7.6) *n* = 227	47.2 (8.7) *n* = 228	−1.1 (−2.3–0.008)	0.052
**Quality of life – mental (SF12 mental component summary score)**^**a c**^				
Baseline	53.4 (5.7) *n* = 304	53.6 (5.4) *n* = 298		
3 months	54.1 (5.6) *n* = 257	54.4 (5.3) *n* = 243	−0.1 (−1.1–0.8)	0.808
6 months	54.2 (5.3) *n* = 238	54.1 (5.1) *n* = 244	−0.7 (−1.6–0.3)	0.185
12 months	54.4 (5.0) *n* = 227	54.2 (4.8) *n* = 228	−0.1 (−1.1–0.8)	0.824
**Physical activity, accelerometer (counts per minute)**^**a**^				
Baseline	239.4 (110.6) *n* = 297	229.7 (103.4) *n* = 288		
6 months	245.5 (111.0) *n* = 230	238.4 (112.9) *n* = 236	0.4 (−14.1–14.8)	0.960
12 months	248.6 (111.0) *n* = 205	245.5 (119.1) *n* = 193	3.2 (−10.7–17.0)	0.653
**Daily steps, measured with accelerometer*(n)***^**a**^				
Baseline	5726 (2425) *n* = 297	5540 (2394) *n* = 288		
6 months	5957 (2653) *n* = 231	5689 (2337) *n* = 236	−105 (− 429–219)	0.525
12 months	5958 (2532) *n* = 205	5916 (2638) *n* = 193	90 (−223–402)	0.572
**Moderate-vigorous physical activity, minutes/day (measured with accelerometer)**^**a c**^				
Baseline	18.9 (17.4) *n* = 297	17.2 (16.8) *n* = 288		
6 months	20.0 (18.1) *n* = 231	17.6 (17.1) *n* = 236	−0.4 (−3.1–2.3)	0.775
12 months	19.8 (18.0) *n* = 205	19.0 (18.6) *n* = 193	0.3 (−2.1–2.8)	0.785
**Total physical activity, hours per week**^**a d**^				
Baseline	33.4 (19.1) *n* = 306	34.3 (19.2) *n* = 309		
3 months	33.4 (19.4) *n* = 260	33.1 (18.1) *n* = 247	−1.1 (−3.6–1.5)	0.412
6 months	31.7 (17.3) *n* = 244	33.0 (17.6) *n* = 250	0.9 (−1.7–3.5)	0.494
12 months	31.3 (16.7) *n* = 229	32.5 (17.6) *n* = 235	0.9 (−1.7–3.6)	0.491
**Planned physical activity (excluding walking), hours per week**^**a c d**^				
Baseline	2.3 (3.8) *n* = 307	2.2 (3.4) *n* = 310		
3 months	2.8 (3.8) *n* = 262	2.7 (3.1) *n* = 251	−0.2 (− 0.8–0.5)	0.575
6 months	2.7 (3.6) *n* = 244	2.4 (2.9) *n* = 250	−0.04 (− 0.7–0.6)	0.895
12 months	2.1 (3.1) *n* = 234	2.1 (3.5) *n* = 237	0.2 (−0.4–0.9)	0.514
**Total walking, hours per week**^**a c d**^				
Baseline	4.7 (5.6) *n* = 307	4.6 (4.8) *n* = 310		
3 months	5.3 (6.7) *n* = 261	5.0 (4.7) *n* = 249	−0.2 (−1.2–0.8)	0.702
6 months	4.5 (4.2) *n* = 242	4.8 (5.1) *n* = 250	0.4 (−0.5–1.3)	0.350
12 months	5.1 (5.6) *n* = 234	5.0 (6.1) *n* = 237	−0.03 (−1.1–1.0)	0.951
**Incidental physical activity (including walking), hours per week**^**a d**^				
Baseline	28.4 (18.2) *n* = 306	29.3 (17.7) *n* = 309		
3 months	27.7 (17.4) *n* = 261	27.4 (16.6) *n* = 246	−0.8 (−3.1–1.6)	0.519
6 months	26.2 (16.6) *n* = 243	28.2 (16.9) *n* = 246	1.5 (−1.0–4.0)	0.245
12 months	25.9 (16.0) *n* = 229	27.3 (16.2) *n* = 235	0.87 (−1.6–3.3)	0.487
**Body Mass Index (BMI), kg/m**^**2**^				
Baseline	28.7 (5.2) *n* = 307	28.4 (5.1) *n* = 309		
3 months	28.1 (5.1) *n* = 243	27.9 (4.8) *n* = 239	0.15 (−0.05–0.4)	0.132
6 months	28.3 (5.0) *n* = 209	28.1 (5.0) *n* = 213	−0.07 (− 0.3–0.1)	0.525
**Short physical performance battery, 0-12**^**a c**^				
Baseline	10.9 (1.5) *n* = 307	10.8 (1.5) *n* = 309		
3 months	11.1 (1.4) *n* = 244	10.9 (1.3) *n* = 239	−0.06 (−0.3–0.1)	0.537
6 months	11.2 (1.4) *n* = 209	11.3 (1.1) *n* = 213	0.17 (−0.04–0.39)	0.108
**Short physical performance battery-continuous summary performance score (CSPS), 0-3**^**c**^				
Baseline	2.52 (0.22) *n* = 307	2.50 (0.23) *n* = 309		
3 months	2.55 (0.23) *n* = 244	2.53 (0.19) *n* = 239	−0.01 (−0.04–0.02)	0.522
6 months	2.56 (0.24) *n* = 208	2.57 (0.17) *n* = 213	0.03 (−0.002–0.06)	0.070
**Sit to stand, time for 5 sit to stands, sec**^**b**^				
Baseline	11.8 (3.5) *n* = 300	12.0 (3.4) *n* = 303		
3 months	11.0 (3.6) *n* = 235	11.4 (3.4) *n* = 234	0.15 (−0.35–0.66)	0.548
6 months	10.4 (3.6) *n* = 200	11.0 (4.2) *n* = 211	0.12 (−0.48–0.73)	0.688
**Gait speed, time to walk 4 m, sec**^**b**^				
Baseline	2.8 (0.7) *n* = 307	2.9 (0.8) *n* = 309		
3 months	2.6 (0.7) *n* = 244	2.8 (0.6) *n* = 239	0.09 (0.003–0.19)	0.043*
6 months	2.7 (0.6) *n* = 208	2.8 (0.6) *n* = 213	−0.01 (−0.1–0.08)	0.819
**Standing balance, sum of feet together, semi-tandem, tandem stance times, sec, 0-30**^**a c**^				
Baseline	29.0 (2.9) *n* = 307	28.7 (3.5) *n* = 309		
3 months	29.3 (2.3) *n* = 243	28.8 (2.9) *n* = 239	−0.19 (−0.71–0.32)	0.461
6 months	29.3 (2.8) *n* = 209	29.5 (1.7) *n* = 213	0.51 (−0.0002–1.0)	0.050
**Single leg stance time, sec, 0-10**^**a c**^				
Baseline	8.4 (2.7) *n* = 259	8.3 (2.8) *n* = 249		
3 months	9.0 (2.3) *n* = 216	8.7 (2.3) *n* = 192	−0.32 (−0.86–0.22)	0.244
6 months	9.2 (2.0) *n* = 185	8.9 (2.3) *n* = 190	−0.21 (− 0.77–0.35)	0.463
**Alternate step test, time for 8 steps onto 18 cm step**^**b**^				
Baseline	8.3 (2.5) *n* = 302	8.4 (2.1) *n* = 302		
3 months	7.7 (2.4) *n* = 236	8.1 (2.1) *n* = 237	0.26 (−0.06–0.58)	0.112
6 months	7.3 (2.1) *n* = 206	7.7 (2.3) *n* = 211	0.23 (−0.11–0.57)	0.189
**Knee extension strength, right leg, kg**^**a**^				
Baseline	16.1 (7.7) *n* = 307	14.6 (6.7) *n* = 309		
3 months	17.4 (7.5) *n* = 244	16.5 (6.6) *n* = 239	−0.55 (−1.72–0.63)	0.361
6 months	17.6 (7.8) *n* = 207	16.2 (7.6) *n* = 211	−0.60 (−1.86–0.65)	0.344
**Knee extension strength, left leg, kg**^**a**^				
Baseline	15.4 (7.4) *n* = 305	14.1 (6.8) *n* = 309		
3 months	16.9 (7.1) *n* = 240	15.8 (7.0) *n* = 239	−0.85 (−2.04–0.34)	0.160
6 months	16.6 (7.1) *n* = 206	15.2 (6.6) *n* = 212	−1.05 (−2.20–0.10)	0.072

^a^Higher scores reflect better performance

^b^Lower scores reflect better performance

^c^Skewed distribution

^d^Self-report measure using the Incidental and Planned Exercise Questionnaire (IPEQ)

^*^Significant outcome

#### Planned sub-group analysis for the primary outcome

In planned sub-group analyses, there was no evidence of statistically significant differential effects of the intervention on the primary outcome of falls by age (*p* = 0.936), sex (male versus female, *p* = 0.680), having fallen in the 12 months prior to baseline (*p* = 0.460) or upper limb dysfunction at entry to the trial, determined by a DASH score > 15 at baseline (*p* = 0.125) [38]. Analysis of the impact within subgroups suggested greater intervention effects in those who had fallen in the past year than those who had not (IRR 0.78, 95% CI 0.41 to 1.51, *p* = 0.462 in those who had fallen 1 or more times, IRR 1.02, 95% CI 0.69 to 1.50, *p* = 0.931 in those who had not fallen in the past year). Further exploratory analyses suggested greater intervention effects in those who had fallen two or more times in the year prior to the intervention (IRR 0.67, 95% CI 0.26 to 1.69, *p* = 0.394 in those who had fallen 2 or more times, IRR 1.00, 95% CI 0.70 to 1.42, *p* = 0.999 in those who fell 0–1 times in the past year) but these differences did not reach statistical significance (*p* = 0.340).

### Adherence with the program

Attendance was recorded at the exercise instruction sessions which were held at weeks 1, 4 and 12. In the intervention group, 294 (96%) participants attended week 1, 260 (85%) attended week 4 and 242 (79%) attended week 12 sessions. In the control group, 298 (96%) participants attended week 1, 260 (84%) attended week 4 and 241 (78%) attended week 12 sessions. In the intervention group, 279 (91%) participants attended two or more exercise instruction sessions. In the control group, 280 (90%) participants attended two or more exercise instruction sessions.

Participants in the intervention group reported completing the exercises less than twice per week, with an average of 94 (SD 63, range 0–287) exercise sessions over the 12 month period. Participants in the control group reported completing the exercises twice per week, with an average of 104 (SD 69, range 0–371) exercise sessions over the 12 month period, as determined by the exercise sessions recorded on the monthly calendars.

### Acceptability of the intervention

Participants completed survey questions on their impressions of the intervention at 3, 6 and 12 months. The total number of participants reporting their impressions varied at different time points. At 3 months 250 out of 261 (96%) intervention group participants felt confident completing the exercise at home. At 6 months, 218 out of 237 (92%) intervention group participants stated that they intended to continue to do the exercises. At 12 months, the mean rating of perceived program benefit for the intervention group was 7.8 out of 10 (SD 2.2). Most participants (*n* = 184/228, 81%) intended to continue to do the exercises, and 218 out of 234 (93%) would recommend the program to other people aged 65 years and older.

At 12 months intervention group participants reported on features they liked about the BEST at Home – lower limb program. The most commonly reported positive features were that the exercises could be done anytime (*n* = 228/230, 99%), the exercises could be done at home (*n* = 221/230, 96%) and that the exercises were simple to follow (*n* = 217/230, 94%). At 12 months, 137 out of 236 (58%) intervention group participants reported that they had problems completing the exercises on a regular basis, with the most common reasons being: going away on holiday (*n* = 50/236, 21%), injury (*n* = 43/236, 18%), lack of motivation (*n* = 40/236, 17%), too busy (*n* = 39/236, 17%), family commitments (*n* = 36/236, 15%) and ill health (*n* = 32/236, 14%).

### Adverse events

One control group participant reported an adverse event associated with the exercise program, which required them to cease the program. Twenty-three participants (11 intervention, 12 control group) reported minor musculoskeletal pains, which were resolved after a short period of time. No fractures occurred while participants were performing the exercises.

## Discussion

Our study did not detect any difference in fall rate between the intervention and control groups. However, it was found that a home-based exercise program for the lower limb can significantly reduce the fear of falling at 3, 6 and 12 months. Gait speed was also significantly faster in the intervention group compared to the control at 3 months. There were no significant differences in physical activity (accelerometer and self-report), quality of life, SPPB, sit to stand, balance, alternate step test, leg strength and gait speed at 6 months.

It is uncertain why there was no clear impact of the exercise intervention on the rate of falls. The exercise program instruction was provided at weeks 1, 4 and 12, and while participants were given suggestions on how to progress the exercise challenge, the exercises were not formally progressed beyond the 12 week time point. This may have resulted in a limited challenge to balance and strength over the 12 month follow-up period. On average participants in the intervention group completed the exercises less than twice per week over the 12 month period, which may not have been a high enough dose of exercise to prevent falls [39]. The intervention group performed the same exercises as the Otago Exercise Programme, which has previously been shown to reduce falls, but the BEST at Home exercise program was delivered in a group-based format and had fewer supervised sessions with the physiotherapist. The original delivery of the Otago Exercise Programme [13, 17] involved more supervision over a 12 month period, with five individual home visits (weeks 1, 2, 4, 8, 26) plus telephone contact when there were no home visits (months 3, 4, 5, 7–12) over a 12 month period. The lower amount of supervision, and particularly individual supervision, offered by the group-based exercise instruction in this study may have resulted in balance and strength challenges which were insufficient to prevent falls. Teaching home exercise in an individual’s home may also be easier as it affords the opportunity to demonstrate where to safely do the exercises, which may lead to increased confidence and therefore more challenging exercise. The original Otago Exercise Programme was also most effective in people aged 80 years and over [40], an older group than the participants in the current study. The Otago Exercise Programme also reported a higher proportion of participants having had a fall at baseline, with 36–56% of participants reporting a fall in the previous 12 months [13, 17, 41], compared to our study with 27% of participants reporting having had a fall in the previous 12 months at baseline. However sub-group analysis suggested greater intervention effects in reducing the rate of falls in those who had fallen in the year prior to baseline assessment than those who had not fallen, and an even greater effect in those reporting two or more falls in the year prior to baseline, although these results did not reach statistical significance.

The significant reduction in fear of falling is an important finding of this study. Fear of falling can lead to restriction of daily activities and is associated with deconditioning, falls and frailty [42]. A recent study [43] found fear of falling to be significantly associated with falls, and a useful index in detecting falls risk in community-dwelling people aged 65 years and over, independent of physical performance. Our results support the findings of a Cochrane Review that found that fear of falling is reduced immediately after an exercise program [44].

The significant difference in gait speed at 3 months is also an encouraging finding, as slow gait speed in older adults has been identified as a risk factor for falls [45]. The improvement in gait speed was not maintained beyond 3 months, perhaps reinforcing the need for greater supervision and/or booster sessions to ensure exercises are appropriately progressed throughout the program to maintain the effects.

The high level of acceptability of the intervention suggests that it is possible to teach people aged 65 years and over to undertake a home-based exercise program with the instruction provided in a group setting. Most participants reported that the exercises were simple to follow and they liked being able to complete them at home at their convenience. However, there was a high proportion of participants reporting barriers to completing the exercises on a regular basis. These barriers included going away on holiday, injuries, lack of motivation, being too busy, having family commitments and ill health. These factors are commonly reported in other studies [46–48] and could be addressed via targeted behaviour change techniques in future projects.

This study had many strengths. It had a pragmatic RCT design, with broad inclusion criteria. It followed the Consolidated Standards of Reporting Trials (CONSORT) guidelines [20] and the protocol was registered prospectively and published [19]. It also attempted to minimise the risk of bias through concealed random allocation to groups, and assessor-blinded outcome assessment. The data for the primary outcome of falls was self-reported by participants, however the person following up primary outcome data with participants was blinded to group allocation and the data was dealt with in a blinded manner. There was a high level of acceptability of the program, with many participants (81% of respondents) indicating that they intended to continue the exercises and 93% would recommend the program to other people aged 65 years and older.

Limitations of the study include a sub-optimal level of adherence to the program that may have precluded participants from reaching the recommended ‘dose’ of balance and strength training required to prevent falls [9, 39, 5]. The participants were a fit and healthy cohort, who self-selected in response to advertisements. The participants appeared to be more active at baseline than the general older population [49] and therefore, the exercises may not have been challenging enough. The exercises were instructed in a group setting, over three sessions at weeks 1, 4 and 12, and there may not have been enough instruction sessions later in the program to allow the exercises to be progressed sufficiently. Attendance at the instruction sessions decreased later in the program and this may have reduced the intensity of balance and strength challenge with which participants were carrying out the exercises, as the later workshops taught participants how to progress the exercises to increase this challenge.

The findings suggest that further research is warranted to establish whether this model of community-based exercise delivery can be enhanced with additional exercise instruction sessions, to provide a greater intensity of challenge to balance and other reminders to promote adherence such as supportive phone calls, text messages, online videos and mobile device apps. There is also a need to investigate targeting of the intervention to participants who would benefit most from an exercise program, such as those with low initial levels of physical activity, poor strength and balance, older age and identified to be at a greater risk of falls. Exercise programs, delivered in a variety of ways, should continue to be offered in the community to assist people meet the recommendations of exercise to prevent falls.

## Data Availability

The datasets used and/or analysed during the current study are available from the corresponding author on reasonable request.

## Abbreviations

BEST: Balance Exercise Strength Training
BMI: Body mass index
CI: Confidence interval
FES-I: Falls Efficacy Scale-International
IPEQ: Incidental and Planned Exercise Questionnaire
IRR: Incidence rate ratio
MVPA: Moderate vigorous physical activity
NSW: New South Wales
SD: Standard deviation
SPPB: Short Physical Performance Battery
WHO: World Health Organisation
